# Influence of protective clothing and masks on facial trustworthiness in an investment game: insights from a Chinese population study

**DOI:** 10.1186/s41235-024-00565-7

**Published:** 2024-06-10

**Authors:** Weiping Wang, Zhifan Li, Xin Lin, Yu-Hao P. Sun, Zhe Wang, Yong Wang

**Affiliations:** 1 Institute of Psychology, Chinese Academy of Sciences, 16 Lincui Road, Chaoyang District, , Beijing, 100101 China; 2 Department of Psychology, University of Chinese Academy of Sciences, Beijing, China; 3 Department of Psychology, Zhejiang Sci-Tech University, Zhejiang, China; 4 The Research Center for Psychological Education, University of International Relations, Beijing, China

**Keywords:** Protective clothing, Mask, Trustworthiness, Investment game, Facial occlusion

## Abstract

Facial features are important sources of information about perceived trustworthiness. Masks and protective clothing diminish the visibility of facial cues by either partially concealing the mouth and nose or covering the entire face. During the pandemic, the use of personal protective equipment affected and redefined who trusts whom in society. This study used the classical investment game of interpersonal trust with Chinese participants to explore the impact of occlusion on interpersonal trust. Faces with moderate initial trustworthiness were occluded by a mask or protective clothing in Experiment 1 and were digitally occluded by a square in Experiment 2, and faces with three levels of initial trustworthiness were occluded by a mask in Experiment 3. Results showed that both undergraduates (Experiment 1a) and non-student adults (Experiment 1b) perceived the faces with protective clothing as more trustworthy than faces wearing standard masks and faces not wearing masks. Faces with the top halves showing were perceived as trustworthy as full faces, while faces with the bottom halves showing were perceived as less trustworthy. The effect of masks is weak and complex. Masks reduced participants’ trust in faces with high initial trustworthiness, had no effect on faces with low and moderate initial trustworthiness, and only slightly increased the trust of undergraduates in faces with moderate initial trustworthiness. Our findings indicate that the lack of information caused by occlusion and the social significance associated with occlusion collectively affect people’s trust behavior in Chinese society. We believe the findings of this study will be useful in elucidating the effects of personal protective equipment usage on perceptions of trustworthiness.

## Introduction

Trust is a crucial factor in human interaction, playing a vital role in determining the success or failure of relationships and transactions (Yamagishi, 2011). The trust cues, which mainly refer to the trustee’s external appearance, such as facial features (Birkás et al., 2014; Ormiston et al., 2017; Todorov et al., 2008;), body language and other nonverbal cues((Penton-Voak et al., 2006)), are important information sources of trustworthiness expectations (Thielmann & Hilbig, 2015). For example, people from different cultures seem to agree that faces with higher inner eyebrows, pronounced cheekbones, wider chins, and shallower noses appear more trustworthy than those with lower inner eyebrows, shallower cheekbones, thin chins, and deep noses (Birkás et al., 2014). During COVID-19, face masks and other personal protective equipment, such as protective clothing, can be an effective non-pharmaceutical intervention against the spread of airborne viruses (Liu et al., 2020; Mniszewski et al., 2014). Face occlusion reduces trust cue acquisition (Freud et al., 2020), making masked faces appear less trustworthy (Bylianto & Chan, 2022; Malik et al., 2021). However, most studies were conducted in Western societies, where mask-wearing is often perceived negatively (Taylor & Asmundson, 2021). Cultural factors may influence trust perception differently in Asia, where mask-wearing is more accepted (Feng et al., 2020). Furthermore, to date, there has been no research investigating the impact of wearing protective clothing on trustworthiness. Using the classical trust game paradigm of interpersonal trust with Chinese participants, the current study examines the effects of face occlusions on interpersonal trust to explore how people form impressions of trustworthiness based on facial appearance.

In modern times, people may cover some parts of their faces for religious and other reasons (Pazhoohi & Kingstone, 2022). For example, some Muslim women may cover their faces by wearing face-covering veils such as the niqab (Kret & de Gelder, 2012). In daily life, it is common occlusion that sunglasses or virtual reality glasses occlude the eye region, while a scarf or a medical mask occludes the mouth region (Kotsia et al., 2008). During the COVID-19 pandemic, masks have become a staple of many people's daily travel attire, and protective clothing has also become a common personal protective equipment for medical staff to prevent respiratory infectious diseases (Liu et al., 2020; Mniszewski et al., 2014). However, masks and protective clothing can reduce the number of facial cues that are available as they partially cover the mouth and nose or completely cover the entire face. This may lead to decreased facial expression identification (Kotsia et al., 2008; Winter et al., 2022) and impair observers' ability to accurately identify the faces of target individuals (Carbon, 2020; Carragher & Hancock, 2020; Winter et al., 2022). Criminals often use facemasks for this reason. Therefore, facial covering practices may impair one’s ability to perceive the expressions and identities of others, potentially diminishing their assessment of others’ trustworthiness.

Wearing face masks can affect our ability to perceive facial signals. This is because masks hide important features of the face, such as the nose, mouth, and cheekbones, which are crucial for evaluating trustworthiness. Santos and Young (2011) found that the face's internal features (eyes, nose, and mouth) provide information that is useful for social inferences, particularly when assessing trustworthiness. In fact, the mouth region plays a significant role in forming impressions of trustworthiness (Vernon et al., 2014). As a result, when the lower half of the face is covered, it can reduce the signal of trustworthiness, which in turn affects the way we judge the trustworthiness of others. Some studies have supported this prediction, showing that people tend to perceive masked faces as less trustworthy and approachable (Bylianto & Chan, 2022; Malik et al., 2021).

How people feel about wearing masks can also affect their trust in masked faces. The COVID-19 pandemic has increased face mask usage globally. However, mask-wearing is less accepted in some countries (Carbon, 2021); in the U.S., it may reduce trust in others, according to a survey (Malik et al., 2021). In Western societies, people may look strange or be judged as strange by others when wearing masks (Carbon, 2021), whereas, in East Asia, mask-wearing is common (Feng et al., 2020), believed to signify positive hygiene practices in Japan (Wada et al., 2012). During the pandemic, wearing masks has been associated with positive social impacts (Klucarova, 2022; Olivera-La Rosa et al., 2020; Perach & Limbu, 2022), freeing individuals from strict isolation (Mniszewski et al., 2014) and promoting participation in social activities (Olivera-La Rosa et al., 2020). Recent studies suggest that masks may enhance perceived trustworthiness (Marini et al., 2021; Oldmeadow & Koch, 2021; Olivera-La Rosa et al., 2020), with masked faces seen as more trustworthy and socially desirable (Olivera-La Rosa et al., 2020). However, studies on trustworthiness perceptions of Asian faces wearing masks found mixed results (Bylianto & Chan, 2022), suggesting uncertainty on how masks influence trust perceptions in Asian cultures.

The COVID-19 pandemic mandated the use of personal protective equipment (PPE) in healthcare settings, obscuring the faces and expressions of clinicians and depersonalizing patient care experiences. When patients cannot identify the clinicians caring for them, they may feel fearful and isolated (Winter et al., 2022). This issue has been observed in certain healthcare areas in the past. However, the utilization of personal protective equipment, particularly protective clothing, during the pandemic has made individuals realize that it acts as both a symbolic and practical barrier between doctors and patients, complicating the establishment of effective communication and connection at various levels (Gács et al., 2021). Therefore, wearing protective clothing may damage trust between medical professionals and patients. Nevertheless, the societal significance behind protective clothing could potentially compensate for the trust impairment caused by obscured faces. In China, individuals wearing protective clothing are called "Baymas" (the protagonist from the movie 'Super Secret Service Team', a warm and positive character). Individuals may hold a high level of trust in them in their daily lives. However, up to this point, no study has examined the level of trust people have in individuals wearing protective clothing.

Trust measurement can be categorized into two types: direct measures and indirect measures (Bauer & Freitag, 2017). Direct measures of trust let participants self-report their trust. Indirect measures attempt to determine trusting expectations by analyzing individuals’ decisions, behavior, and reactions. Previous research on facial trustworthiness has predominantly used direct measures such as the trustworthiness rating. This involves asking participants to rate the trustworthiness of faces using a Likert scale (Birkás et al., 2014; Bylianto & Chan, 2022; Marini et al., 2021; Oldmeadow & Koch, 2021; Olivera-La Rosa et al., 2020). However, this method only evaluates general trustworthiness and does not reveal the mechanism behind trust, nor does it involve specific trust scenarios. The most well-known indirect method of measuring trust is an investment game developed by Berg et al. (1995) and is commonly referred to as the “classical trust game”. The investment trust game is a scenario based on the investment of gold coins, which can reveal people's beliefs about the altruism and reciprocity of others. There is only one study that has used an investment game to assess potential relations between mask usage and perceived trustworthiness (Noah et al., 2021). The results showed that individuals who reported wearing masks more frequently were trusted more than those who reported seldom wearing masks. However, the study only showed the frequency of another person's mask-wearing to participants and not the actual appearance of the person wearing the mask. Therefore, we are not yet clear about the level of trust people have when encountering real masked faces.

In our study, we used the trust game where participants played with an imaginary peer whose face was displayed on the screen. At the beginning of each round, participants had 10 gold coins that they could choose to invest in their peers. If they did, their peer would receive 3 times the amount invested (3N). Participants then had to guess the amount of gold coins that their peers would return to them. In other words, they had to provide an investment behavior and an expectation. Our reason for selecting the economic investment game was due to the display of trust that the game exhibited (Cox, 2004). In this game, a sender would only provide a positive amount in their first move if they trusted that the receiver would return some positive amount after the initial amount sent is tripled. In our study, we assessed participants' trust behaviors based on how much they invested and their trust expectations based on how much they predicted the trustees would repay.

The primary objective of this study was to investigate potential differences in participants' trust behaviors and trust expectations towards individuals wearing masks or protective clothing compared to those not. The study aimed to explore the impact of both the social significance associated with masks and protective clothing and the occlusion of facial features by them on the perception of trustworthiness. To achieve this, we conducted three experiments, manipulating facial occlusion and measuring participants' trust in others using a trust game.

In Experiment 1, faces with moderate initial trustworthiness were occluded by either a standard mask or protective clothing. Protective clothing serves as a good point of comparison to masks. On one hand, in Chinese society, protective clothing carries the same positive social significance as masks but with greater intensity. If the social significance conveyed by the occluding object has a greater impact on facial trustworthiness judgments, then the perceived trustworthiness of faces with protective clothing would be higher than that of faces with masks, and the trustworthiness of faces with masks would be higher than that of uncovered faces. On the other hand, compared to masks, protective clothing provides a greater degree of facial occlusion. It becomes challenging to extract facial features and emotional information from faces that are heavily occluded in this manner. Therefore, if facial trustworthiness judgments primarily rely on the accessibility of facial information, the perceived trustworthiness of faces with protective clothing would be lower than that of faces with masks, and the trustworthiness of faces with masks would be lower than that of uncovered faces.

In Experiment 2, the upper or lower parts of faces were digitally occluded using squares. As squares do not possess any social significance, the results of Experiment 2 can be utilized to examine how the occlusion of facial parts specifically influences the perception of trustworthiness. By comparing the outcomes of Experiment 1 and Experiment 2, we can differentiate the roles played by the occlusion of facial features and social significance in the impact of masks on trustworthiness.

In Experiment 3, faces with three levels of initial trustworthiness were occluded by a mask. These faces were categorized into low trustworthiness, moderate trustworthiness, and high trustworthiness. By comparing participants' trust differences towards these three types of faces, we can gain insights into how the inherent trustworthy cues carried by faces influence the trustworthiness judgments of masked faces. If facial trust cues have an impact on trust judgments of masked faces, we would expect to observe differences in trust judgments among the three types of initial trustworthiness faces. Furthermore, considering that mask occlusion reduces the availability of facial trustworthiness cues, we hypothesize that masking high initial trustworthiness faces will decrease their perceived trustworthiness while masking low initial trustworthiness faces will increase their perceived trustworthiness.

## Experiment 1a

### Method

#### Participants

A total of 75 undergraduate or graduate students (Age range: 18–30; 49 females and 26 males) participated in this experiment. Each participant was paid 5-yuan RMB in cash for their participation, and all of them gave their consent to take part in the study. To detect a main effect of Face Occlusion with a medium effect size (0.25), a power analysis indicated that a sample size of 43 is required to detect at the 0.05 alpha level with a 0.95 power value. The sampling range of previous studies was between 20 and 300 (Bauer & Freitag, 2017; Freud et al., 2020; Kret & de Gelder, 2012; Noah et al., 2021). To safeguard against loss of power due to preregistered participant exclusions and on the basis of the sample size of previous studies, the sample size was increased to 75, which increased power to 100%. This experiment was approved by the Institutional Review Board of Scientific Research Project, Institute of Psychology, Chinese Academy of Sciences (H23065). All methods were performed in accordance with the relevant guidelines and regulations by the Institutional Review Boards.

#### Design

In Experiment 1a, we adopted a 3(Face Occlusion: No Mask = NM, Standard Mask = SM, Protective Clothing = PC) × 2(Trust Stage: Investment vs. Return) within-subject design.

#### Materials

The facial materials included 20 male-neutral faces and 20 female-neutral faces (Ages: 21–40 years old). They were from the face database at Zhejiang Sci-Tech University.

Another group of 62 participants (who did not attend the main experiments) rated the trustworthiness of these 40 faces with a 5-point (1 very untrustworthy, 5 very trustworthy) trustworthiness rating scale. Then six male faces and six female faces with trust scores of about 3 were selected as the stimuli for the experiment (the mean of the trustworthiness rating score was 2.98).

**Fig. 1:**
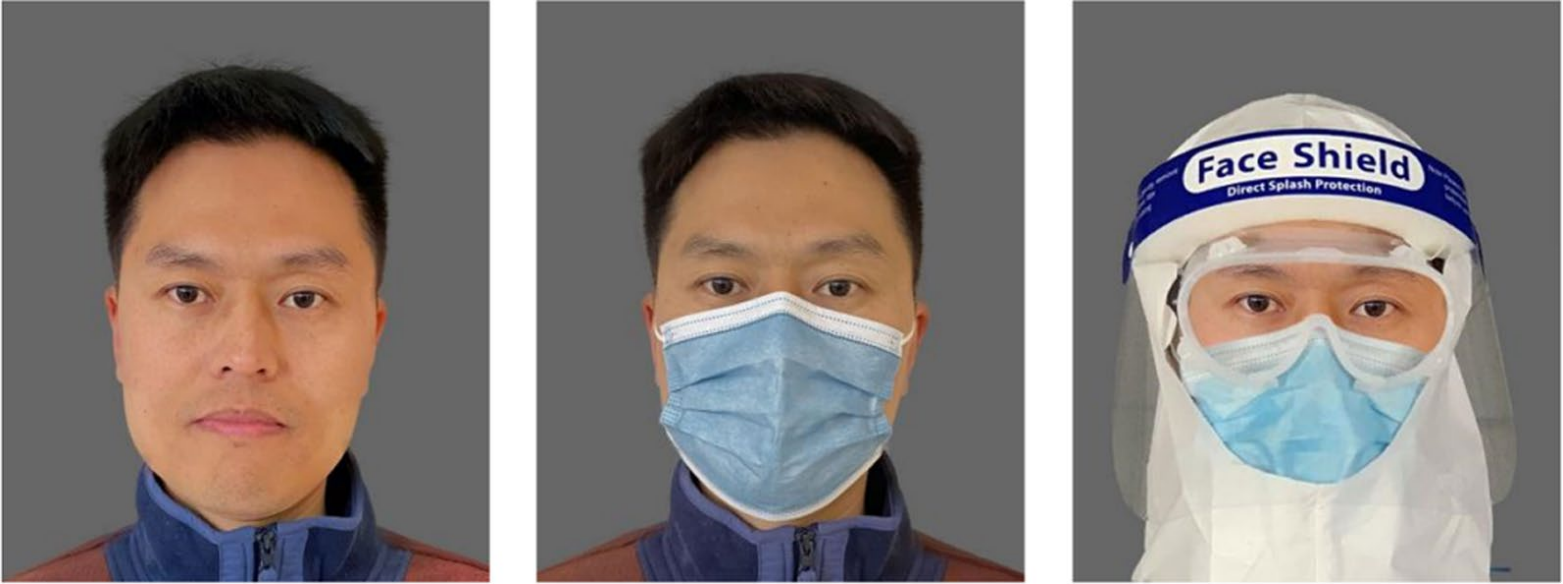
Sample materials for the three Face Occlusion conditions in Experiment 1a, 1b, and Experiment 3 (left and middle)

Photoshop was used to add facial masks or protective clothing to these 12 faces. Figure 1 shows sample faces in the three Face Occlusion conditions. Therefore, we got a total of 36 faces, with one-third wearing masks, one-third without, and one-third with protective clothing. The 36 faces were divided into three sets, each containing 3 (facial mask: no mask = NM, standard mask = SM, Protective Clothing = PC) × 2 (Gender: Male, Female) × 2 identities = 12 faces. All sets were counterbalanced between participants to ensure that faces with the same identity wore masks in one set, did not wear them in another set, and wore protective clothing in the last set. Each participant used only one set of materials during the experiment.

#### Procedure

An online questionnaire application (WenJuanXing, https://www.wjx.cn/) was used to perform the experiment.

The participants played the trust game for 12 rounds as an investor with 12 different trustees after filling out the informed consent form and reading the rules (De Neys et al., 2015; Yu et al., 2022).

In each round, each participant was given a hypothetical sum of 10 gold coins. They were then asked to decide how much of this endowment, ranging from 0 to 10, they wanted to invest in a trustee whose photo appeared on the screen. The investment made by the participant would then be multiplied by three to the trustee according to the rules of the experiment. Once this was done, the participant was asked to estimate the number of coins the trustee would return to her or him (the investor).

The faces in the 12-round game were from three Face Occlusion conditions: NO Mask(NM), Standard Mask(SM), and Protective Clothing(PC). In each Face Occlusion condition, there were 4 faces. The identities of the faces in all three conditions are different, and which faces occurred in which condition was counterbalanced between the participants.

The order of the 12 rounds was randomly selected from three sequences randomly generated for the game by the online questionnaire tool app.

## Results

Two participants who invested zero gold coins in each round were excluded in the data analysis (Table 1).

**Table 1:** Participants’ investment, expected return, and investment income (in coins) in Experiment 1a

		Face occlusion		
		No mask	Standard mask	Protective clothing
Trust stage	Investment	2.9 [2.4,3.3]	3.3 [2.9,3.8]	5.1 [4.4,5.7]
	Expected return	3.9 [3.0,4.8]	4.7 [3.7,5.7]	7.6 [6.1,9.0]
	Investment income	1.0 [0.4,1.7]	1.4 [0.6,2.1]	2.5 [1.5,3.5]

95% confidence interval (CI) in brackets

To investigate the effect of facial occlusion on interpersonal trust, we conducted a 2(Trust Stage: Investment, Expected Return) × 3(Face Occlusion: NO Mask = NM, Standard Mask = SM, Protective Clothing = PC) repeated-measures ANOVA, using the amount of gold coins as the dependent variable.

**Fig. 2:**
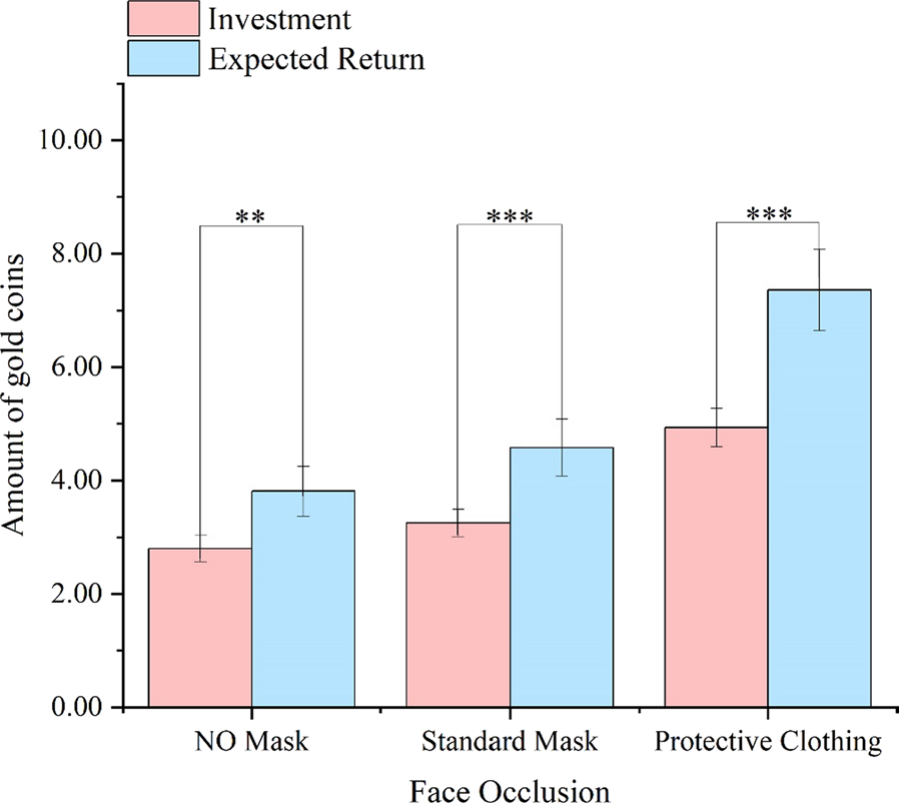
The participants' investment in trustees and their expected return in Experiment 1a with gold coins (Means and SEM). ***p < 0.001,**p < 0.01

The main effect of Face Occlusion was significant,* F*(2,71) = 45.27, *p* < 0.001,η_p_^2^ = 0.39. The SM Faces and the PC faces were more trustworthy than the NM faces (*ps* = 0.001), and the PC faces were more trustworthy than the SM faces (*p* < 0.001), indicating that wearing protective clothing or a standard mask will increase the participants' trust behavior. The main effect of Trust Stage was significant, *F*(1,72) = 20.84, *p* < 0.001,η_p_^2^ = 0.22, suggesting participants believed that the trustees would reciprocate them more. There was a significant interaction between Trust Stage and Face Occlusion, *F*(2,71) = 11.26, *p* = 0.003, η_p_^2^ = 0.16 (Fig. 2).

To examine the difference between investment decisions and expected returns, we conducted pairwise sample t-tests. The results showed that there were significant differences between investment and expected return in the NM condition (*t*(72) = 3.27, *p* = 0.002, Cohen’s d = 0.34), the SM condition (*t*(72) = 3.77, *p* < 0.001, Cohen’s d = 0.40), and the PC condition (*t*(72) = 4.93, *p* < 0.001, Cohen’s d = 0.52). That showed the amount of expected return was higher than the amount of investment in each face occlusion condition. Additionally, we compared the investment income (the difference between investment and expected return) for all three face occlusion conditions. The results showed that the investment income in the PC condition was significantly higher than that in the NM condition (*p* = 0.002) and in the SM condition (*p* = 0.006). The investment income in the NM condition and the SM condition were comparable (*p* = 0.147). That showed that the trustee’s masks didn’t change the participants' trust expectations, but the trustee’s protective clothing increased the participants' trust expectations.

## Experiment 1b

The individuals who participated in Experiment 1a were undergraduates who were more likely to trust others and follow social norms (Henrich et al., 2010). However, this may limit the applicability of their findings. When it comes to economic decision-making, the results obtained from undergraduates do not always align with those obtained from non-student adults (Henrich et al., 2010). To verify the generality of our findings from Experiment 1a, Experiment 1b was conducted using non-student adults across a wide range of ages to investigate the impact of facial occlusion on trust perception.

### Method

#### Participants

A total of 54 non-student adults (Age range: 18–55; 16 females and 38 males) were recruited online. Each participant was paid 5-yuan RMB for participation. All participants consented to taking part in the study. The sample size of Experiment 1b was determined based on Experiment 1a using the same paradigm. To detect a main effect of Face Occlusion with a medium effect size (0.25), a power analysis indicated that a sample size of 43 is required to detect at the 0.05 alpha level with a 0.95 power value. To safeguard against loss of power due to preregistered participant exclusions, the sample size was increased to 54. This experiment was approved by the Institutional Review Board of Scientific Research Project, Institute of Psychology, Chinese Academy of Sciences (H23065). All methods were performed in accordance with the relevant guidelines and regulations by the Institutional Review Boards.

#### Design

In Experiment 1b, we adopted a 3(Face Occlusion: NO Mask = NM, Standard Mask = SM, Protective Clothing = PC) × 2(Trust Stage: Investment vs. Expected Return) within-subject design.

#### Materials, procedure

The materials and procedure of Experiment 1b were the same as those of Experiment 1a.

## Results

One participant who invested zero gold coins in every round was excluded from the data analysis.

To investigate the effect of facial occlusion on interpersonal trust, we conducted a 2(Trust Stage: Investment, Expected Return) × 3(Face Occlusion: NO Mask = NM, Standard Mask = SM, Protective Clothing = PC) repeated-measures ANOVA, using the amount of gold coins as the dependent variable (Table 2).

**Table 2:** Participants’ investment, expected return, and investment income (in Coins) in Experiment 1b

		Face occlusion		
		No mask	Standard mask	Protective clothing
Trust stage	Investment	3.2 [2.7,3.8]	3.5 [2.9,4.0]	4.8 [4.0,5.6]
	Expected return	4.5 [3.4,5.5]	4.8 [3.8,5.9]	7.7 [5.7,9.6]
	Investment income	1.3 [0.6,1.9]	1.4 [0.8,2.0]	2.8 [1.5,4.2]

95% confidence interval (CI) in brackets

**Fig. 3:**
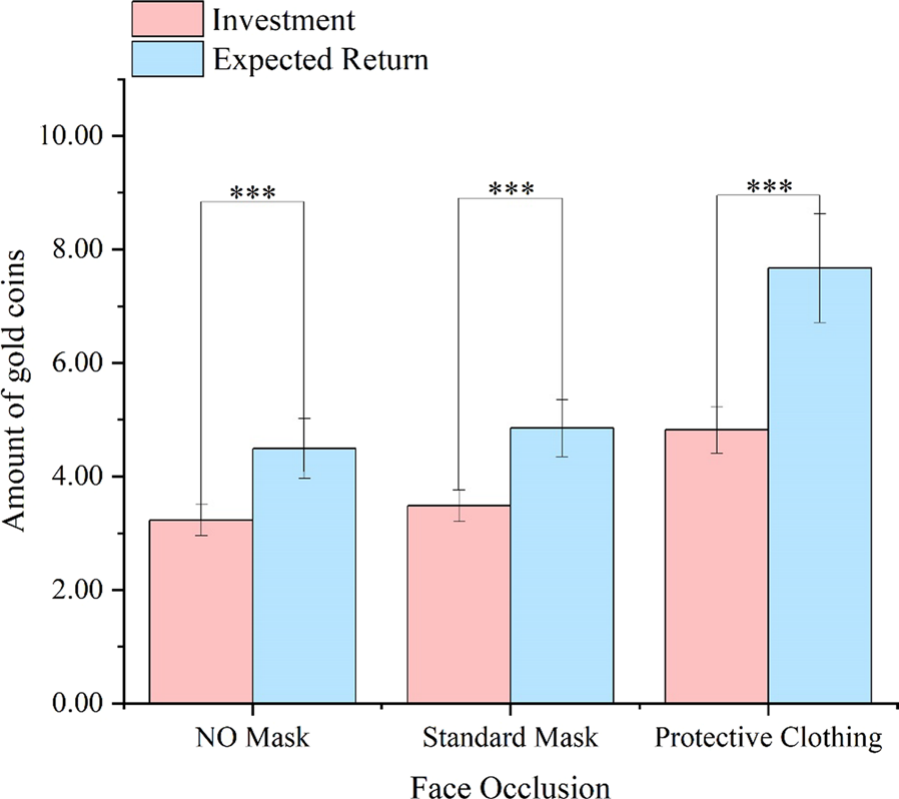
The participants' investment in trustees and their expected return in Experiment 1b with gold coins (Means and SEM). ***p < 0.001

The main effect of Face Occlusion was significant, *F*(2,104) = 12.04, *p* < 0.001,η_p_^2^ = 0.19. The PC faces were perceived as more trustworthy than both the NM faces (*p* = 0.002) and the SM faces (*p* < 0.001). This suggests that consistent with Experiment 1a, wearing protective clothing enhances the participants' trust behavior. However, unlike in Experiment 1a, wearing a mask did not alter the participants' trust behavior. The main effect of Trust Stage was significant, *F*(1,52) = 27.99, *p* < 0.001,η_p_^2^ = 0.35, suggesting participants believed that the trustees would reciprocate them more. There was a significant interaction between Facial Occlusion and Trust Stage, *F*(2,104) = 5.41, *p* = 0.006,η_p_^2^ = 0.09 (Fig. 3).

Similar to Experiment 1a, participants' expected returns were higher than their investments in each face across the NM, SM, and PC conditions (*t*(52) = 3.77, *p* < 0.001, Cohen’s d = 0.42, *t*(52) = 4.53, *p* < 0.001, Cohen’s d = 0.46, *t*(52) = 4.27, *p* < 0.001, Cohen’s d = 0.53). Furthermore, the investment income (the difference between investment and expected) return in the PC condition was significantly greater than that in the NM condition (*p* = 0.029) and the SM condition (*p* = 0.006). The investment income between the NM and SM conditions was comparable (*p* = 0.754). Same as in Experiment 1a, the trustee's masks did not affect participants' trust expectations, whereas the trustee's protective clothing heightened participants' trust expectations.

## Experiment 2

Experiment 1 found that the impact of masks on participants' trust in faces is complex. However, we were uncertain about the degree to which masks influence face trustworthiness by concealing facial trust cues. To investigate the impact of reduced facial information on face trustworthiness, we utilized a neutral digital occlusion in Experiment 2. By combining the findings from Experiments 1 and 2, we aimed to explore the underlying mechanisms of how masks affect face trustworthiness.

### Method

#### Participants

A total of 39 adults (aged 18–40; 21 female) were recruited online. Each participant was paid 5-yuan RMB for participation. To detect a main effect of Face Occlusion with a medium effect size (0.25), a power analysis indicated that a sample size of 36 is required to detect at the 0.05 alpha level with a 0.90 power value. To safeguard against loss of power due to preregistered participant exclusions, the sample size was increased to 39. All participants consented to taking part in the study. This experiment was approved by the Institutional Review Board of Scientific Research Project, Institute of Psychology, Chinese Academy of Sciences (H23065). All methods were performed in accordance with the relevant guidelines and regulations by the Institutional Review Boards.

#### Design

In Experiment 2, we used a within-subject design with 3 levels of digital occlusion (full face showing, top half showing, bottom half showing) and 2 trust stages (investment, expected return).

#### Materials

**Fig. 4:**
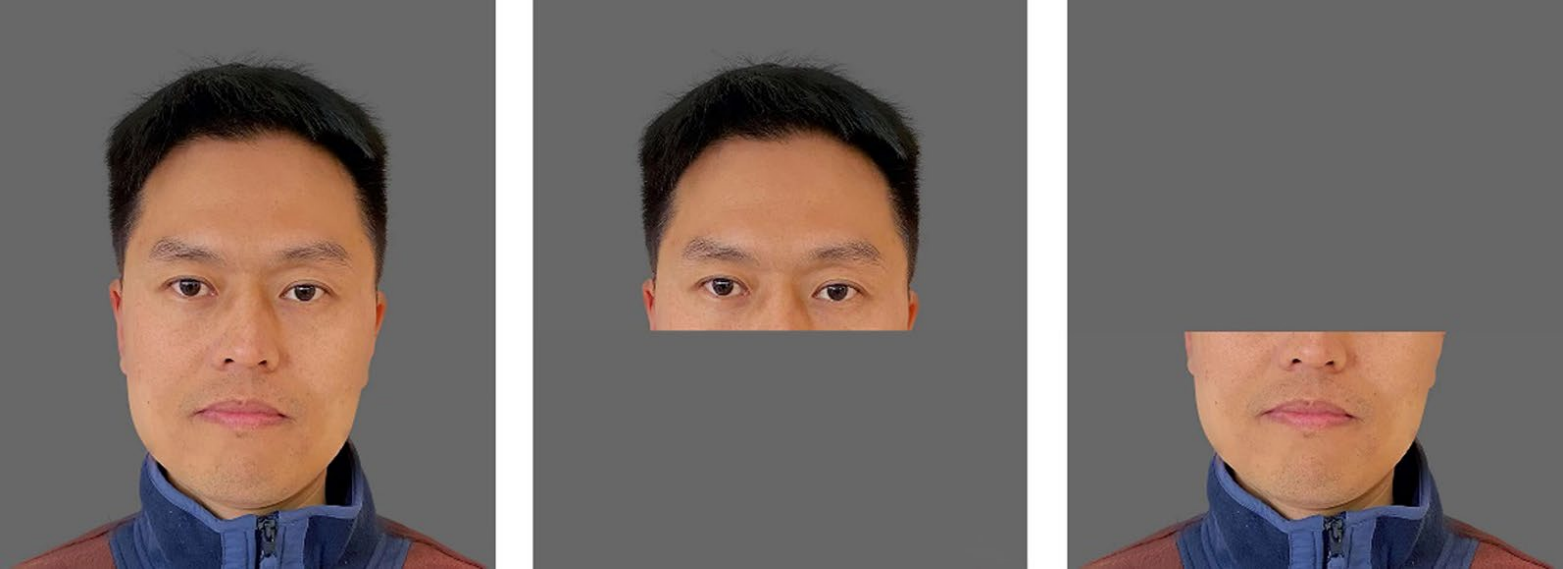
Sample materials for the three Digital Occlusion conditions in Experiment 2

Six male and six female faces from Experiment 1 were split into upper and lower halves, producing 24 images in total (Fig. 4).

#### Procedure

The procedure of Experiment 2 was identical to that of Experiment 1, except for the materials used.

The 18-round investment game involved faces from three different Digital Occlusion conditions: Full Face showing, Top Half showing, and Bottom Half showing. Each condition had 6 faces. The sequence of the 18 rounds was randomly selected from two sequences generated for the game by an online questionnaire tool app.

## Results

One participant who invested zero gold coins in each round was excluded from the data analysis.

**Fig. 5:**
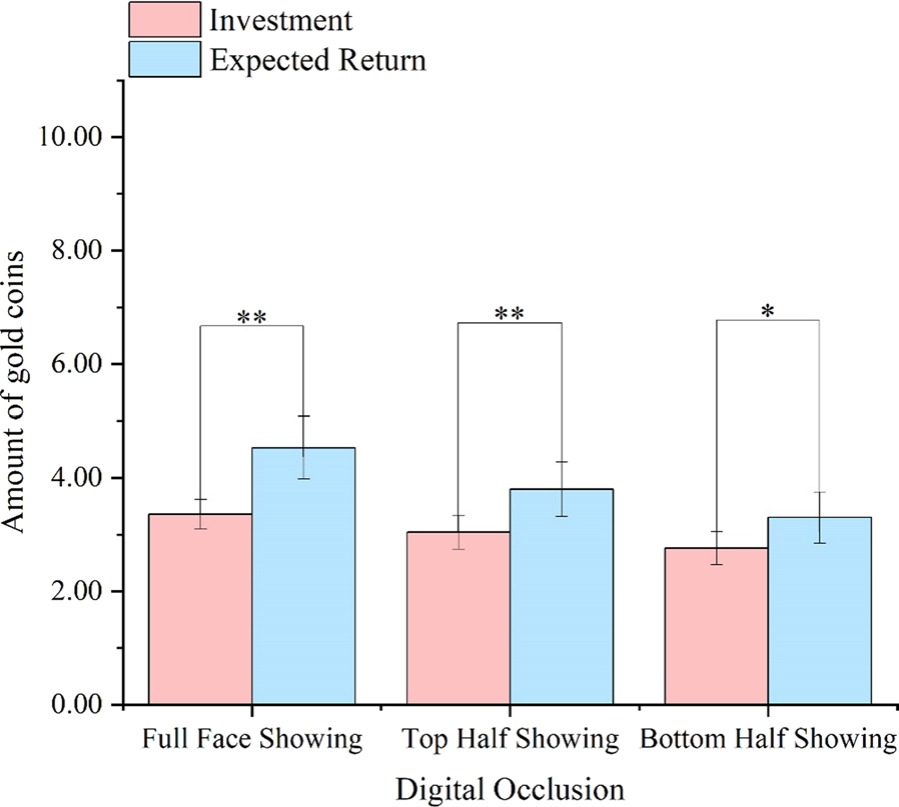
The participants' investment in trustees and their expected return in Experiment 1b with gold coins (Means and SEM). **p < 0.001, *p < 0.05

To examine how digitally covering facial parts affects interpersonal trust, we conducted a 2(Trust Stage: Investment, Expected Return) × 3(Digital Occlusion: Full Face showing, Top Half showing, Bottom Half showing) repeated-measures ANOVA, using the amount of gold coins as the dependent variable (Table 3, Fig. 5).

**Table 3:** Participants’ Investment, Expected Return, and Investment Income ( in Coins) in Experiment 2

		Face showing		
		Full face	Top half	Bottom half
Trust stage	Investment	3.4 [2.8,3.9]	3.0 [2.4,3.6]	2.8 [2.2,3.3]
	Expected return	4.5 [3.4,5.6]	3.8 [2.8,4.8]	3.3 [2.4,4.2]
	Investment income	1.2 [0.4,1.9]	0.8 [0.2–1.3]	0.5 [0.1–1.0]

95% confidence interval (CI) in brackets

The main effect of Digital Occlusion was significant, *F*(2,74) = 7.12, *p* = 0.001,η_p_^2^ = 0.16. The full faces were more trustworthy than the bottom-half showing faces (*p* = 0.001), suggesting the lack of upper facial features will reduce the participants' trust behavior. The main effect of Trust Stage was significant,* F*(1,37) = 9.02, *p* = 0.005, η_p_^2^ = 0.20. There was a significant interaction between Trust Stage and Digital Occlusion, *F*(2,74) = 5.62, *p* = 0.005, η_p_^2^ = 0.13.

There were significant differences between investment and expected return in the Full Face Showing condition (*t*(37) = 3.20, *p* = 0.003, Cohen’s d = 0.49), the Top Half Showing condition (*t*(37) = 2.85, *p* = 0.007, Cohen’s d = 0.49), and the Bottom Half Showing condition (*t*(37) = 2.27, *p* = 0.029, Cohen’s d = 0.34). Furthermore, the investment income (the difference between investment and expected return) in the Full Face Showing condition was significantly greater than in the Bottom Half Showing condition (*p* = 0.012). However, it was comparable to the Top Half Showing condition (*p* = 0.241). This suggests that the absence of top facial features reduces participants' trust expectations. However, obscuring the bottom half of the face did not significantly alter participants' expectations, a finding that diverges from the results of Experiment 1a.

## Comparison between Experiment 1a, 1b and Experiment 2

In Experiment 1a, we observed that participants invested more in faces with masks than in unobstructed faces. However, in Experiment 2, we found no significant difference between faces presented with the upper half showing and unobstructed faces. To test whether there is a difference in the effects of a mask vs. a simple occlusion, we conducted a 2(Trust Stage: Investment, Expected Return) × 3(Experiment: 1a, 1b, 2) × 2 (Occlusion: yes, no), using the amount of gold coins as the dependent variable. Results showed that the main effect of Experiment was not significant, *F*(2, 161) = 0.28, *p* = 0.760. The main effect of Occlusion was not significant, *F*(1,161) = 0.73, *p* = 0.395,. The main effect of Trust Stage was significant, *F*(1,161) = 36.66, *p* < 0.001, η_p_^2^ = 0.20. Importantly, there was a significant interaction between Experiment and Occlusion, *F*(2,161) = 4.12, *p* = 0.017, η_p_^2^ = 0.05. All other effects were not significant (*ps* > 0.127). Further analysis revealed that in Experiment 1a, the investment and expected returns for faces with masks were higher than for unobstructed faces (*p* = 0.008). In Experiment 1b, the difference between faces with and without masks was insignificant (*p* = 0.109); similarly, no significant difference was observed in Experiment 2 (*p* = 0.264). This suggests that among college students, masks can enhance their trust behavior and expectations towards others. This enhancement is derived from the social significance associated with masks rather than the act of occlusion itself, as in Experiment 2, occlusion without social significance did not enhance participants' trust behavior and expectations towards others. However, this enhancement of trust by masks is not stable, as, in Experiment 1b, masks did not enhance trust behavior and expectations of non-college adults towards others.

## Experiment 3

In Experiments 1 and 2, we selected faces with moderate initial trustworthiness as our experimental materials and found that the effect of occlusion on facial trustworthiness was negligible. This might be due to the intermediate strength of the initial trustworthiness of the faces. Previous studies have found that the impact of masks varies depending on the initial trustworthiness of the faces (Marini et al., 2021; Oldmeadow & Koch, 2021; Oliveira & Garcia-Marques, 2022). Therefore, in Experiment 3, we expanded the range of initial facial trustworthiness, choosing faces with low, moderate, and high initial trustworthiness as our experimental materials.

### Method

#### Participants

A total of 30 undergraduate or graduate students (Age range: 18–26; 17 females and 13 males) participated in this experiment. Each participant received compensation of 5-yuan RMB for their involvement and participation and provided their consent to participate in the experiment. For this design (see below), a power analysis indicated that a sample size 28 is required to detect a medium effect size (0.25) at the 0.05 alpha level with a 0.95 power value. The experiment was approved by the Human Research Ethics Committee of Zhejiang Sci-Tech University (202311P002), and all methods were conducted in accordance with the relevant guidelines and regulations by the Institutional Review Boards.

#### Design

In Experiment 3, we adopted a 2(Face Occlusion: No Mask = NM, Standard Mask = SM) × 3(Trustworthiness Level: Low, Moderate, High) × 2(Trust Stage: Investment, Expected Return) within-subject design.

#### Materials

The facial materials include 12 male neutral faces and 12 female neutral faces (Ages: 21–40 years old). They were from the Face database at Zhejiang Sci-Tech University.

A separate group of 20 individuals who were not present during the main experiment was asked to rate the trustworthiness of 100 faces using a 9-point trustworthiness rating scale, where 1 indicated very untrustworthy, and 9 indicated very trustworthy. Based on their ratings, eight faces with low trustworthiness, eight with moderate trustworthiness, and eight with high trustworthiness were selected as stimuli for the experiment. The mean trustworthiness rating score for the low-trustworthy faces was 3.87; for moderate-trustworthy faces, it was 5.07; and for high-trustworthy faces, it was 6.14.

Photoshop was used to add facial masks to these 24 faces. Therefore, we got a total of 48 faces, with half wearing masks and half without. The 48 faces were divided into two sets, each containing 2 (Face Occlusion: No Mask = NM, Standard Mask = SM) × 3 (Trustworthiness Level: Low, Moderate, High) × 2 (Gender: Male, Female) × 2 identities = 24 faces. Both sets were counterbalanced between participants to ensure that faces with the same identity wore masks in one set and did not wear them in the other. Each participant used only one set of materials during the experiment.

#### Procedure

Experiment 3 was programmed by E-Prime 2.0. The participants were seated in front of a screen with a resolution of 1600 × 900 pixels and at a viewing distance of 60 cm. The procedure of Experiment 3 was similar to that of Experiment 1, except for the materials.

In Experiment 3, the participants played a 24-round investment game. The game included two Face Occlusion conditions: No Mask and Standard Mask. Each condition had 4 low-trustworthy faces, 4 moderate-trustworthy faces, and 4 high-trustworthy faces. The order of the 24 rounds was random.

## Results

To investigate the effect of facial occlusion on interpersonal trust, we conducted a 2(Face Occlusion: No Mask = NM, Standard Mask = SM) × 3(Trustworthiness Level: Low, Moderate, High) × 2(Trust Stage: Investment, Expected Return) repeated-measures ANOVA, using the amount of gold coins as the dependent variable.

**Fig. 6:**
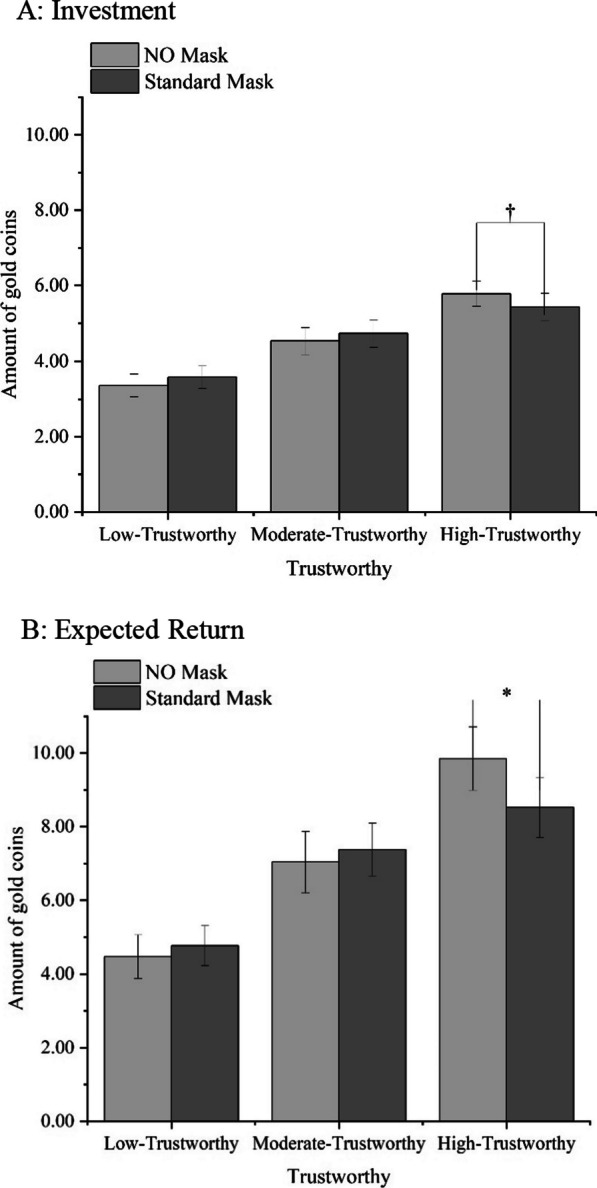
The participants' investment (**A**) in trustees and their expected return (**B**) in Experiment 3 with gold coins (Means and SEM). The asterisks indicate significance level of *p* < 0.10(✝) and *p* < 0.05(*)

The main effect of Face Occlusion was not significant, *F*(1,29) = 0.25, *p* = 0.622. The main effect of Trust Stage was significant,* F*(1,29) = 33.92, *p* < 0.001, η_p_^2^ = 0.54, indicating participants believed that the trustees would reciprocate them more. The main effect of Trustworthiness Level was significant,* F*(1,29) = 51.57, *p* < 0.001, η_p_^2^ = 0.64. The high-trustworthy faces were perceived to be more trustworthy than both the moderate-trustworthy faces and the low-trustworthy faces (*ps* < 0.001). Moreover, the moderate-trustworthy faces were seen as more trustworthy than the low-trustworthy faces (*p* < 0.001). This demonstrates that the perceived trustworthiness of faces impacts participants' trust behavior. It indicates that, even when the bottom half of a face is obscured by masks, the trust cues present in the face continue to exert influence. The interaction between Trustworthiness Level and Face Occlusion was significant, *F*(2,58) = 12.28, *p* = 0.011, η_p_^2^ = 0.14. When it came to high-trustworthy faces, the participants showed less investment and lower expected return on investment in masked faces than in faces without masks (*p* = 0.011). However, for moderate-trustworthy and low-trustworthy faces, the participants showed similar investment and expected returns on investment in both masked and unmasked faces (*ps* > 0.259). The interaction between Trustworthiness Level and Trust Stage was significant, *F*(2,58) = 24.80, *p* < 0.001, η_p_^2^ = 0.46. The interaction between Face Occlusion and Trust Stage was not significant, *F*(1,29) = 1.69, *p* = 0.204. The three-way interaction was significant, *F*(2,58) = 3.94, *p* = 0.025, η_p_^2^ = 0.12 (Table 4, Fig. 6).

**Table 4:** Participants’ investment, expected return, and investment income (in Coins) in Experiment 3

		Low-trustworthy		Moderate-trustworthy		High-trustworthy	
		No mask	Standard mask	No mask	Standard mask	No Mask	Standard mask
Trust stage	Investment	3.4 [2.7,4.0]	3.6 [3.0,4.2]	4.5 [3.8,5.2]	4.7 [4.0,5.5]	5.8 [5.1,6.5]	5.4 [4.7,6.2]
	Expected return	4.5 [3.3, 5.7]	4.8 [3.7,5.9]	7.0 [5.3, 8.7]	7.4 [5.9,8.9]	9.8 [8.1, 11.6]	8.5 [6.8,10.2]
	Investment income	1.1 [0.3,1.9]	1.2 [0.5,1.8]	2.5 [1.4,3.7]	2.7 [1.8,3.5]	4.1 [2.8,5.3]	3.1 [1.9,4.3]

95% Confidence Interval (CI) in brackets

Experiment 3 found that there was a significant difference between the investment and expected return for faces that were perceived as low, moderate, and high in trustworthiness, regardless of whether they wore a mask or not. Across all face occlusion conditions, participants expected trustees to return more gold coins than invested. This expectation was significant for low-trustworthy faces both without masks (*t*(29) = 2.93,* p* = 0.007, Cohen’s d = 0.53) and with masks (*t*(29) = 3.71, *p* = 0.001, Cohen’s d = 0.68), for moderate-trustworthy faces without masks (*t*(29) = 4.52, *p* < 0.001, Cohen’s d = 0.82) and with masks (*t*(29) = 6.32, *p* < 0.001, Cohen’s d = 1.15), and for high-trustworthy faces without masks (*t*(29) = 6.61,* p* < 0.001, Cohen’s d = 1.21) and with masks (*t*(29) = 5.33, *p* < 0.001, Cohen’s d = 0.97).

Additionally, we compared the difference between investment and expected return for NM faces and SM faces. The results showed that for high-trustworthy faces, the difference in the NM condition was significantly higher than that in the SM condition (*p* = 0.018), and for moderate-trustworthy faces and low-trustworthy faces, the difference in the NM condition and the SM condition was comparable (*ps* > 0.669). Experiment 3 yielded results consistent with those of Experiments 1a and 1b. For faces of moderate trustworthiness, the presence of masks did not increase participants' trust expectations. However, Experiment 3 unveiled a new finding: standard masks worn by trustees with high-trustworthy faces reduced participants' trust expectations.

## Discussion

In this study, we used a trust game with Chinese participants to examine the impact of face occlusions on interpersonal trust. Experiment 1 tested Standard Mask (SM) and Protective Clothing (PC); Experiment 2, Digital Occlusion; and Experiment 3, Standard Mask on faces of varying trustworthiness levels. We got three findings: 1) For moderately trustworthy faces, both college students (Experiment 1a) and non-student adults (Experiment 1b) perceived the faces with protective clothing as more trustworthy than faces with and without standard masks. They invested more gold coins in trustees wearing protective clothing than those with or without masks. The investment income (difference between investment decision and expected return) in the PC condition was higher than in the SM and NM conditions. 2) In Experiment 2, participants' investment and investment income were similar in the Full Face Showing and Top Half Showing conditions. However, both were higher in the Full Face Showing condition than the Bottom Half Showing condition, indicating that the absence of upper facial features reduces participants' trust behavior and expectations. 3) The effect of masks is weak and complex. Masks reduced participants’ trust in faces with high initial trustworthiness (Experiment 3), had no effect on faces with low and moderate initial trustworthiness(Experiment 1b and 3), and only slightly increased the trust of undergraduates in faces with moderate initial trustworthiness(Experiment 1a). Our findings indicate that the lack of information caused by occlusion and the social significance associated with occlusion collectively affect people’s trust behavior in Chinese society.

The study found that both college students and non-student adults with a larger age span invested more gold coins in trustees who wore protective clothing compared to those who wore masks or no masks in Experiment 1, indicating that they had more trust in individuals wearing protective clothing. This goes against the speculation that protective clothing reduces facial trustworthy cues and, therefore, decreases facial trustworthiness. Reducing facial cues undermines the accuracy of emotional recognition and the perception of trustworthiness, likability, and intimacy (Pichler & Hemetsberger, 2008). However, our results showed an increase in the trustworthiness of faces wearing protective clothing. This suggests that the social information carried by protective clothing may play a more significant role in people's trust in the faces of protective clothing. During the pandemic, individuals seen wearing protective clothing were frequently healthcare workers or volunteers. In China, people tend to place more trust in them, as demonstrated by the fact that they referred to someone wearing protective clothing as "Baymax", the character in the movie "Super Secret Service Team". Hence, we believe that Chinese people are likely to trust individuals wearing protective clothing more based on their moral judgment of them.

Additionally, in Experiment 1, the participants believed that investing in individuals wearing protective clothing resulted in higher income than those wearing masks or not wearing masks. This further supports our speculation that people's trust in faces wearing protective clothing stems from their moral judgment of them. In the case of interpersonal trust, people are positively related to reciprocity with people who contribute to society (Sweijen et al., 2022). Reciprocity is a response to perceived kind and unkind behavior (Falk & Fischbacher, 2006) and a return for the trust of others (Lahno., 1995). Therefore, in the present study, we observed that the participants gave much more investment to individuals wearing protective clothing than to individuals wearing masks and individuals without masks. At the same time, out of the belief in reciprocity, the participants believe that individuals wearing protective clothing would give them more in return. The findings of protective clothing faces indicate that the social significance associated with occlusion affects people’s trust behavior in Chinese society.

In Experiment 2, participants' investment amounts and investment incomes were comparable in Full Face Showing condition and Top Half Showing condition, but they were higher in Full Face Showing condition than in Bottom Half Showing condition. These findings indicated that the absence of upper facial features is expected to have a negative impact on trust-related behaviors and expectations. A Previous study using high and low trustworthy faces has similar findings (Oliveira & Garcia-Marques, 2022). The discriminability between the perceived trustworthiness of trustworthy and untrustworthy faces was higher when only their top halves were visible compared to when only their bottom halves were visible (Oliveira & Garcia-Marques, 2022). These findings suggest that some facial cues to trustworthiness remain visible when the bottom-half face is occluded and conveys a sufficient signal to make a trustworthy judgment. Previous studies found that besides the mouth region, the eyes region is also relevant for trustworthy inferences (Oosterhof & Todorov, 2008). Based on these findings, we propose that even though internal features such as the eyes, nose, and mouth play a significant role in trust inference (Santos & Young, 2011), for faces partially obscured, the top half has a stronger impact on trust than the bottom.

The study found that masks had varying effects on perceived trustworthiness depending on the initial level of trustworthiness. They reduced participants’ trust in faces with high initial trustworthiness (Experiment 3) but had no impact on participants’ trust in faces with moderate and low initial trustworthiness(Experiments 1b and 2). Our findings align with prior research (Marini et al., 2021; Oldmeadow & Koch, 2021; Oliveira & Garcia-Marques, 2022). These studies used faces with different trustworthiness levels and revealed an increase in perceived trustworthiness for untrustworthy faces when masked, while the effect on trustworthy faces remained inconclusive. These results, along with our findings, suggest that masks make extracting trust signals more challenging, thereby leading to trust judgments that tend towards moderate trustworthiness. The impact of masks on facial attractiveness also exhibits a form of regression to the mean. One study found that unattractive faces appear more attractive when the lower half is masked (Pazhoohi & Kingstone, 2022). Another study corroborates our findings, showing that faces with masks were perceived as less attractive than those without masks, particularly for faces with high attractiveness scores (Kamatani et al., 2021).

The effect of masks on faces with moderate trustworthiness was inconsistent across our three experiments. In Experiment 1a, college students perceived masked faces as more trustworthy, in contrast to Experiments 1b and 3 where this pattern was not observed. Two potential reasons for this disparity could be identified. Firstly, the participant demographics varied, with Experiment 1a involving college students while Experiments 1b and 3 consisted of non-student adults. College students typically exhibit a greater tendency to conform to social norms (Henrich et al., 2010), and during Experiment 1a, wearing masks was a prevalent social norm. This could have influenced college students to trust faces with masks more. Secondly, the timing of the experiments differed. Experiment 1a took place in September 2022 when strict pandemic prevention measures were enforced by the Chinese government. In contrast, Experiment 1b occurred in late November 2022, amidst heated debates over pandemic policies, while Experiment 3 was conducted in 2023 after the restrictions had loosened. These shifts in policies may have impacted perceptions of the social significance of masks. Future studies could explore participants' perspectives on the societal implications of facial coverings, their own mask-wearing behaviors, and trust in masked faces to deepen the understanding of the link between facial coverings and trust.

In this study, we found that participants perceived faces wearing protective clothing as more trustworthy than faces with or without standard masks, specifically for moderately trustworthy faces. It remains unclear whether judgments are based solely on the implications of protective clothing or if unobstructed facial features also play a role. Future research could use protective clothing to cover faces with different levels of initial trustworthiness to investigate the role of facial information in trustworthiness judgments. In addition, research has found that placing a portrait on the outside of protective clothing can help medical personnel connect better with the individuals in their care (Winter et al., 2022). Patients seemed more at ease, and the portraits fostered connection and trust, thereby reducing anxiety and fear and signaling to patients that they were being given holistic, optimal care. Future research could also test the impact of this intervention on facial trustworthiness to explore whether this method can improve potential negative effects caused by the reduction of facial trust cues from protective clothing.

Additionally, as the current study employed a within-subjects design, the effects of faces in protective clothing might have been amplified by participants by making direct comparisons with faces in masks. In future experiments, we could run a between-subjects version, where just one condition is shown to any one person, which might yield different relative effects.

In this study, we found that people perceived faces with protective clothing as more trustworthy than faces wearing standard masks and faces not wearing masks. Masks reduced participants’ trust in faces with high initial trustworthiness but slightly increased or not participants’ trust in faces with moderate and low initial trustworthiness. Our findings indicate that the lack of information caused by occlusion and the social significance associated with occlusion collectively affect people’s trust behavior in Chinese society. As countries slowly recover from COVID-19, policies on epidemic prevention will adapt accordingly, and it remains to be seen whether people’s trust in mask wearers and those wearing protective clothing will evolve. Overall, the pandemic has provided us with an excellent opportunity to explore how changes in the social environment affect and shape people’s trust in one another.

## Data Availability

The datasets used and/or analyzed during the current study are available from the corresponding author upon reasonable request.

## Abbreviations

NM: No mask
SM: Standard mask
PC: Protective clothing
